# Extreme Hypercholesterolemia Following a Ketogenic Diet: Exaggerated Response to an Increasingly Popular Diet

**DOI:** 10.7759/cureus.43683

**Published:** 2023-08-18

**Authors:** Nurit Naveh, Yuval Avidan, Barak Zafrir

**Affiliations:** 1 Department of Family Medicine, Clalit Health Services, Haifa, ISR; 2 Cardiology, Lady Davis Carmel Medical Center, Haifa, ISR; 3 Faculty of Medicine, Technion - Israel Institute of Technology, Haifa, ISR

**Keywords:** low-carbohydrate diet, ketogenic diet, low-density lipoprotein, hypercholesterolemia, hyperlipidemia

## Abstract

A high-fat, very low-carbohydrate diet, often named the “ketogenic diet,” is gaining popularity, particularly among patients with obesity and metabolic syndrome seeking rapid weight loss and improvement in glycemic control. A favorable reduction in triglycerides and an increase in high-density lipoprotein-cholesterol levels is often observed in the ketogenic diet. However, people vary significantly in their low-density lipoprotein-cholesterol (LDL-C) response to the dietary change. Here, we present the case of a 38-year-old normal-weight male with average cholesterol levels showing an extreme fourfold elevation in LDL-C levels, reaching 496 mg/dL after initiating a ketogenic diet. We highlight that a dramatic elevation in LDL-C may manifest following a ketogenic diet in normal-weight people without known genetic dyslipidemias before the dietary change; therefore, increased awareness and close monitoring of blood lipid profile is essential for all individuals following a ketogenic diet. We further discuss the potential mechanisms for the “lean mass hyper-responders” phenotype which has been recently gaining recognition, and suggest that these patients may benefit from ezetimibe therapy, decreasing the absorption of intestinal cholesterol to the liver.

## Introduction

The ketogenic diet, characterized by a very low carbohydrate and high fat consumption, is increasingly being adopted as a nutritional therapeutic measure against obesity and diabetes [1,2]. The diet decreases body weight and triglycerides and blood glucose levels but increases high-density lipoprotein-cholesterol (HDL-C). Although the impact of the ketogenic diet on low-density lipoprotein-cholesterol (LDL-C) is often described as modest, some individuals may experience extreme elevation in their LDL-C level. The mechanisms, safety, and long-term implications of this metabolic reaction are not entirely clear, leading to controversy in the medical community [3,4]. We present a case of extreme hypercholesterolemia following the consumption of a ketogenic diet in a young adult with normal weight and average cholesterol levels before the dietary change.

## Case presentation

**Figure 1:**
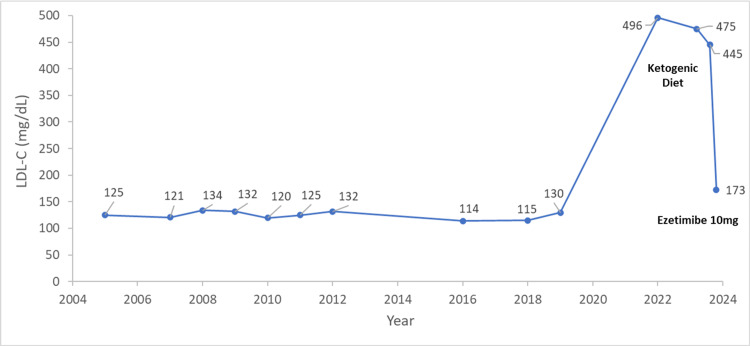
Trend of LDL-C levels over time. LDL-C: low-density lipoprotein-cholesterol

A 38-year-old man was referred for a lipid clinic consultation due to an abrupt onset of extreme hyperlipidemia. He was an active smoker, with a normal body mass index (BMI) of 21.6 kg/m^2^ and no self or family history of cardiovascular disease. Due to intractable gout with arthropathy and hyperuricemia, he was treated with colchicine and a xanthine oxidase inhibitor. Past LDL-C levels during a 15-year period ranged between 100 and 140 mg/dL. A year before the clinic referral he had started following a ketogenic diet, consuming low-carbohydrate, high-saturated fat foods. His LDL-C levels rose dramatically to 496 mg/dL. Figure 1 presents the trend of LDL-C levels over time. Lipoprotein(a) level was normal (40 nmol/L). Blood tests including creatinine, liver enzymes, fasting blood glucose, and thyroid-stimulating hormone were within normal limits. The urine test for proteinuria was negative. One month before the consultation, the patient continued to adhere to the ketogenic diet, and his LDL-C reached 475 mg/dL. He was then advised to change his dietary patterns, lower saturated fat consumption, and initiate a combination lipid-lowering therapy with high-intensity statin and ezetimibe. The patient underwent a CT scan for quantification of coronary artery calcium, receiving a zero calcium score, without any apparent coronary calcifications. After receiving a second medical opinion, and due to the persistence of extreme LDL-C levels in an additional lipidogram (445 mg/dL), the patient decided to initiate a single lipid-lowering therapy with ezetimibe 10 mg and reduce the consumption of saturated fats to some extent. In a few weeks, LDL-C levels rapidly decreased to 173 mg/dL. HDL-C was 70 mg/dL, and triglycerides level was 67 mg/dL.

## Discussion

A very low-carbohydrate, high-fat diet, often named a “ketogenic diet,” restricts carbohydrate intake and drives metabolic changes that are associated with ketosis and a starvation state. Carbohydrates are mostly replaced with fat, often high in saturated fat and cholesterol (though a vegetarian ketogenic diet with the use of unsaturated fatty acids is a possible alternative). The ketogenic diet was developed a century ago as a method to treat intractable epilepsy. However, in the current era of diabetes and obesity epidemics, the ketogenic diet has gained popularity as a dietary approach for achieving rapid weight loss and improvement in glycemic control in patients with prediabetes and diabetes [5].

The effect of a ketogenic diet on blood lipid profile, particularly LDL-C levels, differs significantly across studies [6]. The variability in response is often attributed to the variation in dietary composition, the amount of weight loss, and the duration of the dietary change. In the current case, we present a patient who experienced a fourfold increase in his LDL-C levels following the initiation of a ketogenic diet. He had no apparent additional secondary causes for severe hypercholesterolemia such as medications, severe hypothyroidism, or proteinuria. Lipoprotein(a) was normal, and his lifelong average cholesterol levels before the dietary change did not support monogenic familial hypercholesterolemia. Therefore, the ketogenic diet was the most likely cause of the extreme elevation in cholesterol. Although several studies evaluating the impact of a ketogenic diet on blood cholesterol found only a modest increase in LDL-C levels [6], there are increasing reports in recent years of individuals experiencing a dramatic elevation in their cholesterol level after initiating a ketogenic diet [7]. Interestingly, some reports have suggested that the largest increases in LDL-C levels often occur in patients with a lower BMI and an average pre-diet LDL-C level, labeling these patients as “lean mass hyper-responders” phenotype [8]. A potential mechanistic hypothesis for this relationship, named the “lipid energy model,” suggests that with carbohydrate restriction in lean persons, the increased dependence on fat as a metabolic substrate drives increased hepatic secretion and peripheral uptake of triglycerides contained within very low-density lipoproteins by lipoprotein lipase, resulting in marked elevations of LDL-C and HDL-C, as well as low triglycerides [8]. Our case is in line with these observations as the patient had a low-normal BMI, average baseline LDL-C levels, and an extreme LDL-C response to carbohydrate restriction and high fat consumption.

The long-term impact of a ketogenic diet is unknown and is in dispute, gaining both supporters and antagonists [3]. On the one hand, the ketogenic diet is associated with rapid improvement in metabolic measures such as weight loss, glucose homeostasis, and insulin resistance, as well as a decrease in triglycerides and an increase in HDL-C levels. On the other hand, studies did not show that low carbohydrate diets lead to greater weight loss after a year, with the sustainability and long-term safety of the ketogenic diet being unknown [3,4]. Moreover, in those with extremely elevated LDL-C levels in response to a ketogenic diet, a prolonged increase in the consumption of cholesterol and saturated fat is believed to be associated with the development of atherosclerosis, as epidemiologic, observational, and genetic studies support a causal association between LDL-C and atherosclerotic cardiovascular disease. However, interestingly, it was suggested that the ketogenic diet is often associated with a positive change in LDL subfractions, with predominant large buoyant particles that may be less atherogenic, in contrast to small dense LDL particles that are associated with metabolic syndrome and increased cardiovascular risk. Nevertheless, the total concentration of apolipoprotein B particles increases. In addition, it should be noted that a similar pattern of LDL subfractions is often observed in patients with familial hypercholesterolemia, who face an increased risk of atherosclerotic cardiovascular disease if drug treatment is not initiated at a young age.

In this case, the patient decided to undergo a CT scan showing the lack of coronary calcifications. However, it is important to point out that the assessment of calcified plaque development by non-contrast-enhanced CT scan is limited at a young age, as the vast majority of individuals under 40 years of age will have a coronary calcium score of zero. Therefore, imaging for subclinical atherosclerosis such as coronary artery calcium scoring is limited in its ability to risk stratify patients and guide therapeutical decisions at this age.

A rapid decrease in cholesterol level was observed less than two months after initiating ezetimibe, with a 65% decrease in LDL-C from peak levels. Extreme LDL-C response to the ketogenic diet may be associated with cholesterol hyperabsorption, and therefore the use of ezetimibe, which inhibits NPC1L1 protein and decreases the transport of intestinal cholesterol to the liver, may produce a far more potent effect on LDL-C reduction in patients on a ketogenic diet than in the general population. Nevertheless, additional mechanisms may be associated with a hyper-response to saturated fat intake, such as those mediated through sterol-responsive element-binding protein, affecting LDL receptor expression on hepatocytes, and, accordingly, a positive response to statin therapy is also expected.

The national dietary recommendations for the prevention of cardiovascular diseases advise that a ketogenic diet may be considered for a short term of up to one year for a reduction in weight and rapid improvement in metabolic measures (class of recommendation IIb, level of evidence A). In the absence of long-term data, it is recommended to avoid the ketogenic diet beyond a one-year period (class of recommendation III, level of evidence C) [9]. Professional consensus documents additionally recommend that patients with known dyslipidemias should avoid ketogenic diets [6]. However, as presented in this case, extreme hyperlipidemia following a high-fat, very low-carbohydrate diet may also manifest in people with an average lipidogram before the dietary change, and therefore, lipid profile should be reassessed periodically in all patients following a ketogenic diet [10].

## Conclusions

Extreme hypercholesterolemia may develop as a result of a ketogenic diet and is suggested to occur more often in people with lean body mass. Increased awareness and close monitoring of blood lipid profile are essential for all individuals following a ketogenic diet, as people vary significantly in LDL-C response to the dietary change, and extreme elevation may also occur in patients without known dyslipidemias. Further research is required to understand the mechanisms and genetic variants associated with the variable interindividual response of blood cholesterol to the ketogenic diet, its long-term safety, and the future impact on the development of atherosclerotic cardiovascular disease.
